# Dr. Smith on Benzoic Acid in Urinary Disorders

**Published:** 1842-09-03

**Authors:** John Smith Soden

**Affiliations:** Surgeon to the United Hospital, Bath


*On Benzoic Acid in Urinary Disorders.* By John Smith Soden, Surgeon to the United Hospital, Bath.—In the last volume of the Medico-Chirurgical Transactions, there is a paper by Mr. Ure on gouty concretions, in which he states that most unequivocal proofs have been afforded him of the efficacy of benzoic acid in correcting and removing certain disordered states of the urine in individuals prone to attacks of gravel. In the "Provincial Medical and Surgical Journal," of February 16, 1843, Dr. Walker, of Huddersfield, published an account of the advantage he had witnessed from the use of benzoic acid, combined with balsam of copaiba, in certain affections of the urinary organs. Dr. Walker's statements induced me to adopt his practice, and the first favourable opportunity of testing its efficacy occurred a few days after I had read Dr. Walker's paper, when I was summoned to an elderly gentleman who had long suffered from irritable bladder and enlarged prostate. Three years ago I saw this patient on account of retention of urine. I was then informed that, for a considerable time, he had frequent inclination to pass urine, though able to void only a small quantity at each call, and that the urine was generally loaded with mucous secretion. I found enlargement of the prostate, but had no difficulty in passing a catheter; I emptied his bladder, and the urine drawn off contained a considerable quantity of muco-purulent deposit. The catheter was passed daily, and the bladder washed out with warm water; the hip-bath, with rest, and the means ordinarily adopted in such cases, soon mitigated the severity of this attack. The patient acquired the power of introducing the catheter himself, and has used the instrument, I believe, daily ever since that period. I occasionally felt the instrument strike against a calculus, but the state of the prostate, and advanced age of the individual, readered an operation inadvisable. During the three last years he has taken most of the remedies generally recommended on such occasions, and thinks the uva ursi has been most serviceable to him. He had not, for a long time, been under the care of a medical man, but trusted entirely to his own management, till I was sent for in March last, in consequence of aggravation of suffering. He showed me the urine he had recently passed and drawn off. It deposited a large quantity of muco-purulent discharge. He complained much of the irritability of the bladder. I injected warm water, and, as on former occasions, he had derived more benefit from the exhibition of uva ursi, than from any other remedy, I prescribed that medicine, together with the use of the hip-bath, and a suitable regimen; as no material relief ensued at the end of three days, I directed the benzoic acid in the following form:—Benzoic acid, one drachm; Balsam of copaiba, half an ounce; Yolk of egg, enough to form a mixture with seven ounces of camphor mixture. Two tablespoonfuls to be taken thrice a-day.

I never witnessed anything equal to the efficacy of this medicine; the urine became clearer after the first dose, and in two days it was perfectly free from mucous deposit; the irritability of the bladder was lessened, and in four days the patient resumed his self-management. I did not feel the calculus during this attendance. The gentleman left Bath about six weeks after this period. I saw him a few days before his departure; he told me that he was as well as usual, that he continued to use the catheter, but that the urine was quite clear, and that when he observed any tendency to mucous deposit he had recourse to the mixture, and always with success.

The result of this case induced me to give the medicine a trial at the United Hospital, and our intelligent house surgeon, Dr. Morgan, has been kind enough to give me the heads of four cases in which it has been exhibited at that institution.

*Case I.*—A man aged thirty-five, applied for admission as an out-patient, complaining of frequent desire to make water, which has existed for the last month; the urine deposits mucous sediment; the patient has no gonorrhœa, and refers his disorder to being much exposed to cold and wet. On passing a catheter the urethra was found perfectly natural, but there was some slight hæmorrhage after the urine had been evacuated; has some pain in the loins; pulse rather strong; was at first cupped on the loins, and ordered aperients; and then diosma and the pareira brava, with opiates, were given in succession. After having attended for three weeks, he complained of some pain in the joints, for which he was ordered colchicum, and though it greatly relieved the rheumatic affection, produced no beneficial effect upon the state of the bladder. Mr. Soden saw him, and directed the mixture, with benzoic acid and balsam of copaiba. He found benefit after using it for two days, and in ten days was perfectly well.

*Case II.*—A married woman, apparently in good health, was admitted as an out-patient, stating that she had frequent desire to make water; the urine depositing on cooling (she says) a whitish sediment; urine slightly acid; she has been under medical treatment at intervals during the last six months, but without deriving any benefit from the means adopted. The mixture, with benzoic acid and balsam of copaiba, was ordered immediately, and she was discharged, cured, in three weeks.

*Case III.*—A man, aged fifty, has been under the care of two surgeons for a month, owing to having suffered from irritability of the bladder. He has now frequent desire to make water; a small quantity of blood is occasionally passed with the last drop of urine; some ropy mucous is deposited in the urine, which is slightly acid, though it very soon becomes ammoniacal on standing; there is some irritation at the glans penis; on sounding, no stone could be detected. He was ordered the benzoic acid mixture, but only continued his attendance for three visits (eight days,) during which time great relief was afforded, and as he has not since applied at the hospital, he is most probably well.

*Case IV.*—A man, aged thirty-seven, after a severe attack of gonorrhœa, which appeared, by his description, to have been attended with acute inflammation of the bladder, was admitted an out-patient. He complains of being obliged to make water very frequently, having to get up six or eight times in the night to empty his bladder; has much pain in front of the pubis; some ropy mucous is deposited in the vessel, after the urine has been standing some time. After trying several other remedies without advantage, the benzoic acid mixture was ordered, from the use of which he experienced great relief in two or three days, and at the end of ten days no mucous was discovered in the urine.

The most remarkable circumstance connected with the exhibition of this medicine, as far as my experience goes, is its decided efficacy in diminishing, and in some instances, completely suppressing the muco-purulent deposition in the urine, which is so prominent a symptom in most cases of affection of the bladder. I am aware that a doubt may be very fairly entertained whether this effect is to be attributed to the benzoic acid, or to the balsam of copaiba, or to their combination. In the few cases which I have just related I was induced to give both of these medicines, from the advantage which had been derived from their use in Dr. Walker's practice. It is, however, very desirable to ascertain the effect of benzoic acid alone in similar cases, more particularly as balsam of copaiba so frequently disagrees with delicate stomachs. I shall therefore not fail to avail myself of future opportunities of trying it, uncombined with other active ingredients; and the hope of inducing my brethren of the Association to endeavour to establish the real worth of a remedy that promises to be serviceable in a very formidable and distressing class of complaints, has been my principal motive in occupying so much of the time of the meeting.—*Prov. Med. Journ.*, *July* 30.

